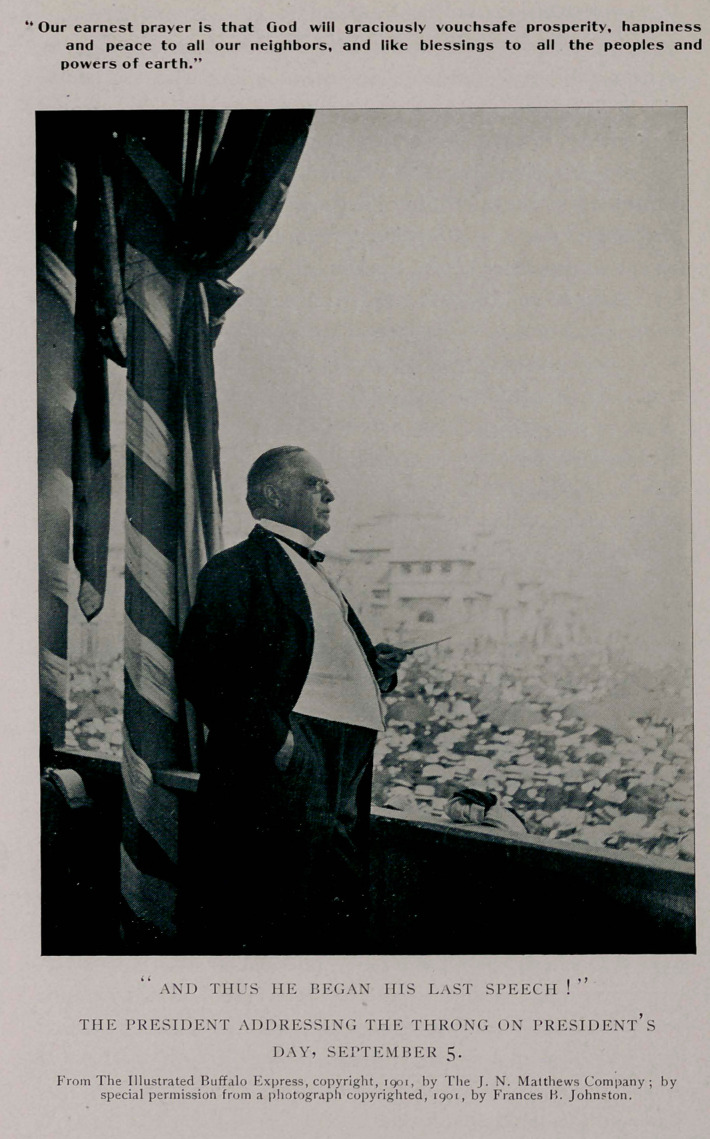# The Assassination of President McKinley

**Published:** 1901-10

**Authors:** 


**A Monthly Review of Medicine and Surgery.**

*EDITOR:*

WILLIAM WARREN POTTER, M. D.

All communications, whether of a literary or business nature, books for review and exchanges should be addressed to the editor: 284 FRANKLIN STREET, BUFFALO, N. Y.

VOL. XLI.—LVII. OCTOBER, 1901. NO. 3.

### The Assassination of President McKinley.

PRESIDENT WILLIAM McKINLEY, twenty-fourth chief magistrate of the Republic, was mortally shot in the Temple of Music on the grounds of the Pan-American Exposition at Buffalo, at seven minutes after four o'clock, Friday afternoon, September 6, 1901, and died from the effects of wounds then and there inflicted, Saturday, September 14, at fifteen minutes after two o'clock in the morning. His assassin, Leon Czolgosz, was immediately secured and has been tried, convicted, and condemned to death for the crime in the courts of Buffalo.

In these brief sentences is recorded the most heinous, dastardly, revolting murder ever committed within the boundaries of this free and enlightened union of states. In making this assertion we do not forget that two other Presidents,—Lincoln and Garfield,—also have suffered death in a similar manner. But we also remember that the immortal Lincoln was killed during a period of civil war when the passions of men ran riot, and when there was great bitterness of feeling toward the man who had saved the Republic from disruption.

When Garfield was shot there was a high party feeling throughout the land and many were disappointed in the distribution of offices. There were factions at war and the quarrel ran high. A fanatic seized upon this moment to revenge a fancied wrong by killing an innocent and well-meaning executive.

WILLIAM MCKINLEY

Twenty-fourth President of the United States.

Born January 29, 1843—Died September 14, 1901.

From The Illustrated Buffalo Express, copyright, 1901, by The J. N. Matthews Company; by special permission from a photograph copyrighted, 1900, by Frances B. Johnston.

But when William McKinley was so cruelly assailed there was peace everywhere within our broad land, and there were neither party nor partisan quarrels. Moreover, the good President had borne himself so well in his great office that even his party opponents respected him. There were no animosities toward him in the ranks of the opposition, while within, thanks to his tactful management, his own party was a unit in the support which it accorded his administration.

The circumstances attending his taking off were so cruelly inhuman as to lend an additional sadness to the tragedy. This great President came to Buffalo as the guest of the Pan-American Exposition on a mission of good-will to the American people, with peace, prosperity and progress as the settings of a picture that must portray forever a classic epoch in American history. These three essentials to national greatness were the themes of his gifted speech made on the exposition grounds on the morning of September 5, when amidst the assembled thousands he delivered to his fellow-countrymen a message that will take rank with the greatest of state papers, and which attracted the attention of the civilised world.

There was joy that day in Buffalo; the inhabitants and visitors assembled in vast numbers to see the President and listen to his words of wisdom. He was surrounded by his cabinet, by high officials of state, by the diplomatic corps, and by distinguished citizens who had gathered from remote places to do homage to the Chief and lend dignity to the occasion. The sun was shining in all its resplendent glory on as beautiful a scene as the eye need view. The exposition buildings and the landscape architecture reflected the warm effulgent rays of a fading-summer sun which could light up no more charming spectacle in all the world. The turn stiles at the gates were kept busy registering the people who came, and by night-time the total was 116,000, a larger number than had attended in a single day since the opening of the fair. The people were in holiday attire and flags were flying from every staff and pinnacle, making a panorama of life and beauty rare to behold.

**"Our earnest prayer is that God will graciously vouchsafe prosperity, happiness and peace to all our neighbors, and like blessings to all the peoples and powers of earth."**

"AND THUS HE BEGAN HIS LAST SPEECH!"

THE PRESIDENT ADDRESSING THE THRONG ON PRESIDENT'S DAY, SEPTEMBER 5.

From The Illustrated Buffalo Express, copyright, 1901, by The J. N. Matthews Company; by special permission from a photograph copyrighted, 1901, by Frances B. Johnston.

The President, too, was happy. He was enjoying a respite from the cares of an office to which he had been twice chosen by seventy millions of people. He was mingling with the people who had thus honored him, and was ready to render an account of his stewardship. He was taking the only real vacation he had enjoyed since his first election. His devoted wife had recovered, or nearly so, from a grievous illness and she, too, was lending her gracious presence with smiling countenance to the enchanting scene. And thus he began his last speech!

A review of the military at the Stadium followed the address, and then the luncheon at the New York State Building, with 200 of his suite and other guests,—civic, diplomatic and military; next a card reception at the Government building; then a return to the Milburn home, that had been given over to the President during his visit, where dinner was served; afterward a drive to the exposition to view the illumination of the tower and buildings; and, finally, the fireworks were witnessed,—the most costly and magnificent pyrotechnic display that ever had been offered, which more than 100,000 people enjoyed. A return to the Milburn house in the balmy evening air at 10 o'clock concluded the ceremonies. And so ended the last happy day the President was to enjoy on earth! Let us hope, let us believe, it was the happiest in all his presidential life.

Early next morning the presidential party was astir, for this was to be a day at the Falls of Niagara, and then a public reception and some informal visiting of the exhibits and other attractions at the exposition. The special train went direct to Lewiston, where awaiting trolleys were boarded and the gorge was viewed as the cars slowly wended their way along the river. At the International Hotel, Niagara Falls, a modest luncheon was served lasting but an hour, then the power-house and other points of interest were visited, after which the return to Buffalo was made in season for the public reception that had been appointed to begin in the Temple of Music at four o'clock. On reaching the exposition grounds a short visit was paid to the mission house.

Precisely at the time appointed the President took his place and began to take each one by the hand with all his well-known cordiality. In the next seven minutes a large number had been received, when lo! a man approached with his right hand enveloped in a white handkerchief, giving the impression that he was disabled. The President attempted to grasp the left hand which the assassin offered; but the villain, pushing it aside, at the same time planted his right against the President's breast and discharged twice in rapid succession a pistol which was concealed beneath the handkerchief.

A scene of consternation followed which cannot be described in calm words. Amidst shoutings, faintings, and swayings of the crowd the President was tenderly assisted to a chair in which he calmly sat without murmur or one word of reproach. Meanwhile, before the fiendish assailant could discharge the weapon again he had been secured and taken away. The ambulance had been summoned, and on its arrival the President was laid on the stretcher-bed which, with its precious burden, was lifted into the vehicle. Mr. Cortelyou, the President's faithful secretary, Mr. John G. Milburn, president of the exposition, and the hospital internes took their places in the ambulance which was then driven to the hospital.

---

The medical history of this saddest of events is told elsewhere in the JOURNAL by those who are familiar with the facts. Dr. Parmenter's account of the operation will be read with interest. His competency as a surgeon is unchallenged, and especially has he had considerable experience with gunshot wounds of the abdomen. Why he was not continued as a member of the staff during the after-treatment of the President is not explained.

Dr. Wilson tells, in graphic manner, the details of the case from beginning to end, and his interesting narrative is a word-picture that has rarely been equaled in medical literature.

It is not our purpose to deal with the surgical aspects of this startling and awful tragedy in this place. It is sufficient for us to remark that there is little in the management to criticise, while there is much to commend. We leave the long-distance critics a clear field for the exercise of their talents.

Buffalo is too deeply grieved that she has become even the innocent cause of the President's death, to pay heed to idle or unkind remarks concerning the physicians who so creditably performed their parts, and who did all that human skill could do to save a life so precious to the nation.

In the midst of so much that tends to sadden our hearts, it is some consolation to reflect that men were easily accessible so thoroughly competent to deal with the case. It must ever be a matter of local pride that a suitable hospital had been provided within the exposition grounds, where such an important operation could be done quickly; and, further, that three of our surgeons could be assembled promptly, who had the skill and presence of mind to deal adequately with such formidable wounds, without a moment's unnecessary delay. These two facts, we repeat, are sources of much comfort to the medical profession of Buffalo, and ought to take precedence in the minds of the people over every other consideration.

We cannot be personal, or make invidious distinctions here, but to all the surgeons and physicians who served in the President's case our gratitude is tendered. Each played his part well; all share the honor of having used a combined skill and judgment rarely equaled and never excelled, and none should be exalted or belittled above or below a common meed of praise that each alike is entitled to receive.

---

A word of praise likewise may be said of the nurses, hospital corps men, and other attendants who so devotedly served the wounded and dying President during those trying days and nights. The newspaper men in the camp opposite Milburn house also were self-sacrificing and faithful under many difficulties.

We are greatly indebted to the *Illustrated Buffalo Express*, for courteously supplying to the JOURNAL the illustrations which we publish as a part of the history of the case.

## Figures and Tables

**Figure f1:**
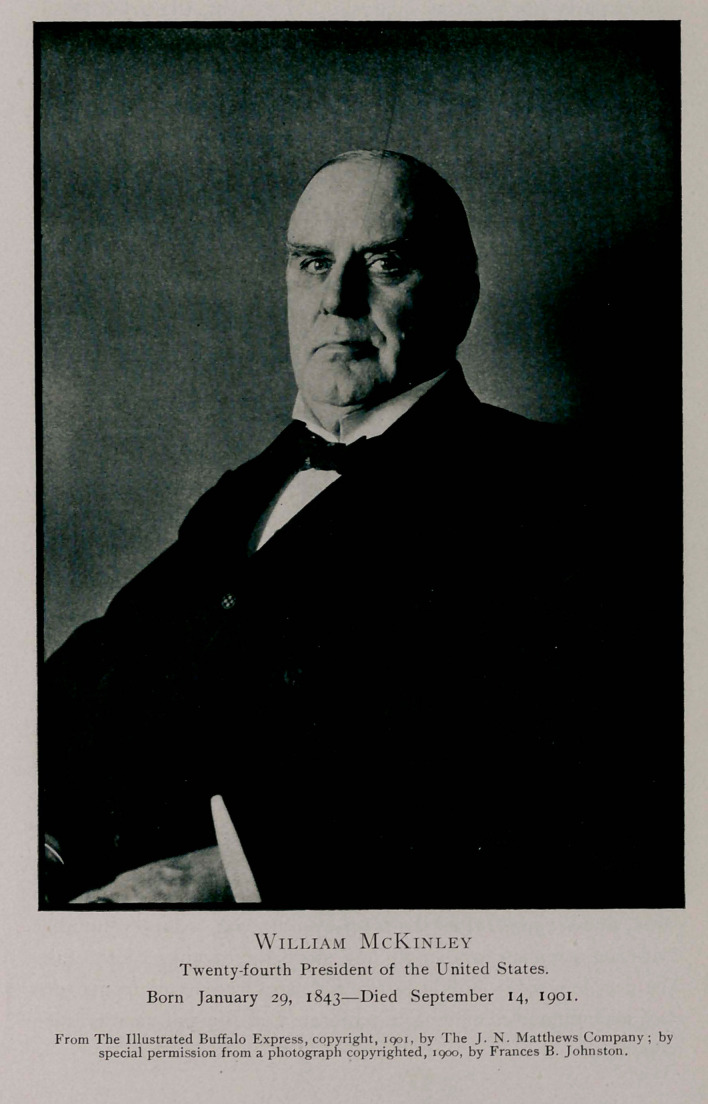


**Figure f2:**